# Efforts towards the institutionalisation of evidence-informed decision-making

**DOI:** 10.1136/bmjebm-2024-112962

**Published:** 2025-12-01

**Authors:** Laura Boeira, Emily Hayter, Sandy Oliver, Laurenz Mahlanza-Langer, Donald Simeon, Mukdarut Bangpan, Veronica Osorio Calderon, Ludovic Reveiz, Shelly-Ann Hunte, Firmaye Bogale Wolde, Tanja Kuchenmuller

**Affiliations:** 1 Instituto Veredas, Belo Horizonte, Brazil; 2 On Think Tanks Consulting, London, UK; 3 EPPI-Centre, Social Science Research Unit, UCL Social Research Institute, University College London, London, UK; 4 Pan-African Collective for Evidence, Johannesburg, South Africa; 5 Caribbean Centre for Health Systems Research and Development, The University of the West Indies, St. Augustine, Trinidad and Tobago; 6 Department for Evidence and Intelligence for Action in Health, Pan American Health Organization, Washington, DC, Columbia, USA; 7 Knowledge Translation Directorate, Ethiopian Public Health Institute, Addis Ababa, Ethiopia; 8 Research for Health Department, Science Division, World Health Organization, Geneva, Switzerland

## Efforts towards the institutionalisation of evidence-informed decision-making

Evidence-informed decision-making (EIDM) refers to the systematic and transparent process of identifying, appraising and mobilising the best available evidence to inform the development of safe and effective health policies and programmes.^1^ This approach has been applied across various contexts, such as improving health outcomes for Indigenous communities in Brazil, reforming alcohol legislation in Moldova and updating national treatment policy for drug-resistant malaria in Uganda.^2 3^ EIDM is not only a practical necessity for addressing public health challenges but also a moral imperative grounded in the principles of the respect for persons, responsibility and accountability. However, there remains a critical need for continuous support from local stakeholders and international funders to facilitate the institutionalisation of EIDM. This practice is understood as both a process and outcome, involving (re-)creating, maintaining and reinforcing norms, regulations and standard practices necessary for evidence to become a routine part of health policy-making.^4^

The importance of EIDM in shaping global health policies and practices has gained considerable recognition, particularly as many countries experimented with and created new ways of applying evidence to policy-making during the COVID-19 pandemic.^5^ EIDM also plays a critical role in achieving the Sustainable Development Goals. In response to this growing importance, the Evidence-informed Policy Network (EVIPNet), a key initiative of WHO to increase country capacity in accessing and using the best available evidence, issued a call for action in 2021,^6^ highlighting the need to strengthen the institutionalisation of EIDM structures and processes that are demand-driven, ethical and multidisciplinary.

With this analysis, we aim to examine international efforts to strengthen the evidence ecosystem for health decision-making. By reviewing a range of tools, partnerships and strategies employed by international organisations, NGOs, think tanks and government agencies, we highlight both the differences and commonalities among these approaches. We explore how they complement one another to enhance the integration of evidence into health policy-making worldwide.

## Guiding countries towards the institutionalisation of EIDM

Over time, organisations have developed frameworks, guidelines and materials to help countries advance their EIDM efforts. Table 1 summarises tools, partnerships and strategies identified by a WHO ad hoc technical group on EIDM institutionalisation.

The WHO checklist supporting the routine use of evidence during the policy-making process outlines six critical domains, each containing key actions that are essential to EIDM institutionalisation.^7^ The domains include governance; standards and routinised processes; leadership and commitment; resources and capacity-building/strengthening; partnership, collective action and support; and culture. The checklist is designed to assist countries to strengthen their evidence ecosystems. For example, within the Governance domain, initial steps include conducting a situation analysis or proof of concept, as well as collaborative priority-setting, establishing a preliminary institutional knowledge translation mechanism and discussing a clear legal frame along with government mandates and responsibilities.

In the domain of Standards and Routinised Processes, actions include familiarising stakeholders with international tools and creating an environment that supports institutional memory and routine practices. Further, under the Partnership, Collective Action and Support domain, promoting networking, experience sharing, stakeholder engagement and relationship-building among national champions and stakeholders facilitate national momentum for EIDM.

Building trust by providing leaders with solid research and policy-making skills is a concrete action within the Leadership and Commitment domain. A foundational step under the building Resources and Capacity domain involves assessing stakeholders’ capacity to find and use research evidence, while also raising awareness among donors about the importance of fostering EIDM in their work. Finally, a key activity within the Culture domain is to initiate public campaigns that emphasise the value of research for citizens, generating interest in the use of evidence both in policy and in their daily lives.

Globally, several key initiatives are actively working towards institutionalising EIDM to strengthen decision-making processes, especially within public health and socioeconomic policy. These initiatives offer frameworks, tools and activities designed to build stronger evidence ecosystems. In this section, along with table 1, we explore these initiatives, highlighting their goals, strategies and efforts to foster collaboration, enhance leadership and improve governance in EIDM. Furthermore, we highlight where each initiative aligns with the six domains of the WHO EIDM checklist.

### WHO EVIPNet and the Evidence for Policy and Practice Information Centre (UK)

The EVIPNet and the Evidence for Policy and Practice Information Centre’s (EPPI-Centre’s) efforts in countries such as Trinidad and Tobago and Tajikistan employ the *WHO EIDM checklist* to map out institutionalisation stages and ensure that EIDM becomes a more sustainable practice at both national and local levels. The WHO checklist enables stakeholders to assess the state of their evidence ecosystem, providing them with a pathway to integrate EIDM into their broader governance framework.

### Brazilian Coalition for Evidence (Brazil)

The Brazilian Coalition for Evidence employs both the WHO EIDM Institutionalization Checklist and the *Rapid Evidence Support Systems Assessment*.^8^ These tools aim to strengthen evidence units within Brazil’s public administration by mapping the types of evidence that are mainstreamed into the decision-making process and supporting efforts to institutionalise EIDM. By supporting EIDM, the Brazilian Coalition plays a vital role in mainstreaming evidence within the country’s policy apparatus and enabling more informed governance.

The *EPPI-Centre* has investigated the six domains of institutionalisation in two studies. An *autoethnography* of policyrelevant research and research-informed policy-identified key mechanisms of change as outward-looking and inclusive, learning partnerships. A study on institutionalising EIDM in Latin America, with an emphasis on Brazil, revealed cultures of open government practices and consolidating institutional memory as important contextual factors. These studies provide a nuanced understanding of how EIDM evolves in different contexts, offering valuable suggestions for other countries seeking to enhance their own EIDM frameworks.

### Ethiopian Public Health Institute—Knowledge Translation Directorate (Ethiopia)

The Ethiopian Public Health Institute is applying the *WHO EIDM Institutionalization Checklist* to develop its roadmap for establishing Health Technology Assessment (HTA) in Ethiopia. This initiative seeks to recognise the need for improved coordination and legal mandates for EIDM structures within government agencies to guide national health policies. The Institute is establishing a new HTA-related unit at the Ministry of Health, the Policy, Strategy and Research Lead Executive Office.

### Pan-African Collective for Evidence (South Africa)

Pan-African Collective for Evidence (PACE) employs the *Evidence Mapping*^9^ and the *Evidence Management Guide*^10^ developed by South Africa’s Department of Planning, Monitoring and Evaluation. These formalised tools support the institutionalisation of EIDM across various sectors within South Africa. PACE’s work focuses on central government coordination and capacity building for EIDM, ensuring that decision-makers have the tools and training required to use evidence effectively. By formalising the tools and introducing frameworks on evidence use (eg, the evidence-informed policy-making appendix in the National Policy Development Framework), PACE is strengthening the country’s ability to make evidence-informed decisions.

### Overseas Development Institute (UK)

Overseas Development Institute (ODI), in collaboration with South Africa’s Department of Environmental Affairs, has developed *Guidelines and Good Practices for Evidence-Informed Policy*^11^ through the Building Capacity to Use Research Evidence programme. The guidelines aim to foster a culture of evidence use within South Africa’s environmental sector. Through its partnerships and capacity-building initiatives, ODI has contributed significantly to the use of evidence in environmental policymaking. Their work includes formalising approaches to strengthening evidence use within government departments and fostering a more evidence-driven culture.

### Caribbean Centre for Health Systems Research and Development (Trinidad and Tobago)

The Caribbean Centre is working on strengthening the capacity for evidence use in public health systems through training, coaching and fellowship programmes, in collaboration with WHO and government ministries. This initiative is committed to embedding EIDM within Trinidad and Tobago’s public health policies by establishing links with the Ministry of Health and other social sector ministries. By promoting knowledge translation, the centre is ensuring that public health policies are grounded in highquality evidence.

### Southeast Asia Evidence Policy and Partnership Network (Thailand)

Southeast Asia Evidence Policy and Partnership Network (SEAEPP) focuses on building capacity for evidence synthesis and institutionalising EIDM within Thailand. The network employs the *WHO EIDM checklist* to assess the needs and gaps in the country’s evidence ecosystem. In partnership with higher education institutions and government departments, SEAEPP delivers workshops to help academics and policymakers generate and use evidence to inform national policy. Their pilot of the WHO checklist allows stakeholders to identify areas of improvement and strengthen the role of evidence in governance in the health and education sectors.

### International Network for Advancing Science and Policy

The *Context Matters Framework*^12^ developed by International Network for Advancing Science and Policy (INASP) and Politics & Ideas, is used to diagnose opportunities for enhancing evidence use in government agencies. The framework has been piloted with government departments in countries such as Ghana and Peru. This framework has been adapted to support regional programmes in South Asia and East Asia Pacific, as well as to enhance EIDM practices in countries such as Uganda and Pakistan.

### Strengthening Evidence Use for Development Impact

Strengthening Evidence Use for Development Impact’s (SEDI’s) *Political Economy Plus (PEA+) methodology*^13^ combines political economy analysis, knowledge systems analysis and organisational analysis to identify entry points for embedding EIDM within national sectors. SEDI has conducted analyses across nine sectors in three countries—Pakistan, Ghana and Uganda—with support from ODI and INASP. Their work has produced comprehensive country reports, which are used to advance EIDM at the national and sectoral levels.

## Context-specificity of EIDM institutionalisation

The examples above highlight efforts to promote and institutionalise EIDM within specific contexts, involving partnerships and collaborations with various organisations, such as academic institutions, research centres and government agencies. These activities—ranging from workshops, knowledge translation, evidence mapping, developing roadmaps and establishing new evidence units—are context-specific and tailored to meet local needs. Capacity building and training activities are central to these efforts, alongside assessment and evaluation of the state of EIDM and evidence support systems.

In many countries, assessments of evidence support systems serve as an initial step in EIDM institutionalisation. These assessments are typically combined with an analysis of the broader national political context. By engaging researchers and other stakeholders in providing in-depth analyses and promoting co-design processes with policymakers, these tools, partnerships and strategies can drive organisational change and inform the implementation of diverse activities to strengthen evidence use.

## Looking to the future: a call for funders

For countries to fully explore and benefit from the process of EIDM institutionalisation, continuous support is required from both local stakeholders and global funders. By prioritising EIDM and investing in initiatives that promote evidence use, funders can help strengthen decision-making processes and improve policy outcomes. In recognition of the need for cross-border learning and the creation of synergies and collaboration, a Global Coalition for Evidence was launched at the Global Evidence Summit in September 2024 by WHO and its partners. The Coalition will formally assume the work of WHO’s ad hoc technical group on EIDM institutionalisation, leveraging the exchange of experiences and methodological approaches, while creating strong partnerships and synergies across the evidence ecosystem.

In the short term, funders can support the refinement of existing methods and tools and their piloting in selected countries. Over the longer term, they can support the horizontal scaling-up of proven approaches through collaborative learning and coordinated efforts. Additionally, funds are needed to empower countries that are at the forefront of efforts, pioneering EIDM to strengthen their domestic evidence-support systems and further embed EIDM sustainably in their national context. This process requires meaningful engagement between policy-makers, civil society, evidence intermediaries and evidence producers to create a lasting impact.

## Figures and Tables

**Table 1:** Initiatives, tools and strategies illustrating overlapping domains for EIDM institutionalisation

Organisation	Tools/authors	Aims	Activities/partners	Domains of institutionalisation
WHO EVIPNet and the Evidence for Policy and Practice InformationCentre (EPPI- Centre) (United Kingdom of Great Britain and Northern Ireland, UK)	WHO Evidence-informed Decision-making (EIDM) Institutionalisation Checklist (2022)/WHO	▸ To pilot the WHO EIDM Institutionalisation Checklist ▸ To explore the principles of EIDM in relation to instIitutionalisation	▸ Assessing evidence ecosystems, identifying institutionalisation stages, exploring key actions and evaluating tool usefulness ▸ Refining the protocol, conducting situational analysis, identifying institutionalisation stages and activities ▸ Sites: Trinidad and Tobago, Tajikistan ▸ Scoping review	This is a tool to inform planning for enhancing institutionalisation, taking into account **all six domains**. The scoping review covers all **six domains** of institutionalisation.
Brazilian Coalition for Evidence (Brazil)	WHO EIDM Institutionalisation Checklist/ (2022)/WHO Rapid Evidence Support Systems Assessment (RESSA—2023)/McMaster Health Forum	▸ Strengthening units within public administration	▸ Mapping evidence units, types of evidence mainstreamed and supporting EIPM efforts ▸ Assessing evidence ecosystems and identifying institutionalisation stages	This activity assessed and enhanced organisational **governance**, **standards/routinised procedures**, **partnerships**, **leadership and commitment**, and **resources** for supporting EIDM.
The EPPI-Centre (UK)	Autoethnography (2023)/EPPI Centre Evidence Mapping/EPPI Centre WHO EIDM Institutionalisation Checklist (2022)/WHO	▸ To identify mechanisms of change ▸ To identify EIDM actors and understand EIDM institutionalisation in Brazil ▸ To assess progress in institutionalisation	▸ Conducting autoethnographic research ▸ Identifying key stakeholders and analysing the social and political factors influencing the institutionalisation of EIDM in Latin America with a case study of Brazil ▸ Piloting the WHO checklist in a range of countries	Two studies investigated the **six domains** of institutionalisation and their external environment. (1) An autoethnography revealed how collaborative **partnerships** have been key mechanisms for developing capacity in terms of human resources and ways of working. (2) Investigating Latin America revealed **cultures** of open government practices and consolidating institutional memory as important contextual factors.
Ethiopian Public Health Institute — Knowledge Translation Directorate (Ethiopia)	WHO EIDM Institutionalisation Checklist (2022)/WHO	▸ Recognising the need for coordination and legal mandates for EIDM units/ structures	▸ EIDM ecosystem assessment, integrating the WHO checklist, developing a roadmap for HTA institutionalisation, establishing a new unit ▸ Engaged in developing new evidence champions	Developing new evidence champions and recognising the need for coordination and legal mandates for EIDM structures spanned the domains of **governance**, **leadership and commitment**, **resources** and **partnerships**.
Pan-African Collective for Evidence PACE (South Africa)	Evidence Mapping (2016)/ Department of Planning Monitoring and Evaluation - Republic of South Africa Evidence Management Guide (2021)/Department of Planning Monitoring and Evaluation— Republic of South Africa	▸ Supporting EIDM institutionalisation in South Africa	▸ Areas of work: centre of government coordination support, capacity building, formalisation of tools, introducing chapters of evidence use	PACE advanced support for EIDM institutionalisation by spanning the domains of **partnership**, **resources** and **standards/routinised procedures.**
Overseas Development Institute (ODI) (UK)	Guidelines and Good Practices for Evidence Informed Policy in a Government Department (2016)/ODI and Department of Environmental Affairs of the Republic of South Africa	▸ Guidelines that underpin an evidence-informed approach to policy-making within a department or line ministry—covering external and internal factors	▸ Partnership with the Department for Environmental Affairs South Africa through the Building Capacity to Use Research Evidence (BCURE) programme (VakaYiko Consortium) resulting in an organisation-wide approach to strengthening evidence use in the sector	ODI’s guidelines and good practices enhance **standards/routinised procedures** and **partnerships.**
Caribbean Centre of Health Systems Research and Development (Trinidad and Tobago)	WHO EIDM Institutionalisation Checklist (2022)/WHO	▸ Strengthening capacity for evidence use through training, coaching and fellowship programmes	▸ Knowledge translation, establishing linkages between academia and government ministries ▸ Ministry of Health and Social Sector Ministries in Trinidad and Tobago ▸ Capacity building for EIDM at local academic institutions	This work combined human **resources** and **partnerships** across government ministries and between government and academia.
Southeast Asia Evidence Policy and Partnership Network (SEAEPP) (Thailand)	Evidence synthesis WHO EIDM Institutionalisation Checklist (2022)/WHO	▸ Capacity building for evidence synthesis and evidence used to support the institutionalisation of EIDM. ▸ Assessing the needs and gaps of EIDM in Thailand	▸ Engaging with higher education institutions and government departments including Ministry of Education to build capacity for conducting evidence synthesis to inform decisions ▸ Delivering workshops for academics and policy-makers to generate and use evidence to inform decisions ▸ Piloting the WHO checklist	SEAEPP is strengthening **partnerships** and human **resources,** and windows of opportunity (**culture**).
Politics & Ideas and International Network for Advancing Science and Policy(INASP) (UK)	Context Matters Framework (2016)/INASP and Politics & Ideas	▸ Participatory process in partnership with government agencies to diagnose windows of opportunity for strengthened evidence use across 6 domains of external context and organisational context	▸ Piloting evidence diagnostics with the Ghana Environmental Protection Agency and the Peru Secretariat of Public Administration resulting in diagnostic reports and co-developed change plans with each agency ▸ Using the diagnostic tool to identify windows of opportunity for improved evidence use in UNICEF regional programmes in South Asia and East Asia Pacific resulting in co-developed diagnostic reports and prioritisation of actions for change ▸ Adaptating and using the tool with the Office of the Prime Minister in Uganda and National Tarriff Commission in Pakistan via SEDI (below). Framework also adapted as a research/analytical tool (eg, Using Evidence in Africa framework (with PACE) and Evidence in African Parliaments (with the African Centre for Parliamentary Affairs (ACEPA))	This work focuses on **partnerships** and windows of opportunity (**culture**).
Strengthening Evidence use for Development Impact (SEDI) programme (UK)	Political economy analysis plus (PEA+) methodology (2021)/ SEDI Programme	▸ Combines elements of PEA, knowledge systems analysis and organisational analysis to identify entry points for embedded use of evidence in sectors at national level	▸ Conducted across nine sectors in three countries by the Sustainable Development Policy Institute SDPI in Pakistan, the Africa Center for Economic Transformation ACET in Ghana and the Economic Policy Research Centre EPRC in Uganda with support from ODI and INASP as part of the analysis phase of the SEDI project funded by the Foreign, Commonwealth & Development Office FCDO. Combined reports for each country are available via Oxford Policy Management OPM	This work focuses on windows of opportunity (**culture**) for embedding use of evidence nationally.

## Key Messages

⇒ Institutionalising evidence-informed decision-making (EIDM) is a growing field of interest, yet there is limited understanding how to effectively assess, plan, monitor and evaluate its stages of development.

⇒ The study enhances our understanding of the diversity of tools and approaches available to assess EIDM institutionalisation, illustrating their application in settings around the world, and highlighting how they overlap and complement each other.

⇒ The findings directly support researchers, policy-makers and practitioners in assessing and identifying their country’s stage of EIDM institutionalisation, as well as guiding actions to foster the routine use of evidence in policy-making.
